# Tracking and appraising maternal and perinatal death surveillance and response implementation in Nigeria: a historical timeline and policy analysis

**DOI:** 10.1186/s12978-025-02196-7

**Published:** 2025-11-26

**Authors:** Fatimat M. Akinlusi, Uchenna Gwacham-Anisiobi, Donald Imosemi, Oluwarotimi I. Akinola, Adedoyin Ogunyemi, Brenda Isikekpei, Victoria Egunjobi, Kikelomo O. Wright, Adeyemi Okunowo, Ndubuisi Ezumezu, Rachel A. Thompson, Bosede B. Afolabi, Aduragbemi Banke-Thomas

**Affiliations:** 1 Department of Obstetrics and Gynaecology, Lagos State University College of Medicine, Ikeja, Lagos Nigeria; 2 Faculty of Epidemiology and Population Health, London School of Hygiene and Tropical Medicine, London, WC1E 7HT United Kingdom; 3 Centre for Clinical Trials, Research, and Implementation Science, College of Medicine, University of Lagos, Idi Araba, Lagos Nigeria; 4 Lagos State Maternal Perinatal Death Surveillance and Response Technical Committee, Lagos State Ministry of Health, Ikeja, Lagos Nigeria; 5 Department of Community Health and Primary Care, College of Medicine, University of Lagos, Idi Araba, Lagos Nigeria; 6 Department of Community Health and Primary Health Care, Lagos State University College of Medicine, Ikeja, Lagos Nigeria; 7 Department of Obstetrics and Gynaecology, College of Medicine, University of Lagos, Idi Araba, Lagos Nigeria

**Keywords:** Maternal death, Perinatal death, Stillbirth, MPDSR, Verbal autopsy, Social autopsy, Nigeria

## Abstract

**Background:**

Nigeria bears one of the highest global burdens of maternal and perinatal mortality, despite decades of global and national efforts to address preventable deaths. Maternal and Perinatal Death Surveillance and Response (MPDSR) is a key strategy to reduce preventable deaths. This study synthesises the historical trajectory and policy evolution of MPDSR in Nigeria, examining factors shaping its uptake, scope, and institutionalisation.

**Methods:**

We conducted a historical timeline and policy analysis using systematic review principles. Peer-reviewed and grey literature were retrieved through comprehensive database and web searches, complemented by stakeholder engagement. Data were extracted from 24 eligible documents and analysed using Walt and Gilson’s policy triangle framework to explore policy content, context, actors, and processes over time.

**Results:**

Four phases of MPDSR implementation were identified, showing a shift from facility-based maternal death reviews to broader inclusion of perinatal and community components. Federal policymakers and professional associations have driven national-level adoption, while state-level uptake varies depending on political will and capacity. Community and facility-based MPDSR have evolved as fragmented practices rather than an integrated system. Facility-level implementation is comparatively more established, while community-based MPDSR remain limited, donor-driven, and inconsistently integrated. Progress towards institutionalisation is hampered by weak legal frameworks and insufficient subnational capacity. Persistent challenges faced by frontline workers and variable community engagement further undermine MPDSR sustainability.

**Conclusion:**

To maximise the potential of MPDSR as a tool for accountability and system strengthening, Nigeria must integrate community and facility-based surveillance within a unified system, backed by legislation, sustained financing, and capacity building.

**Supplementary Information:**

The online version contains supplementary material available at 10.1186/s12978-025-02196-7.

## Abstract (plain language summary)

In Nigeria, many pregnant women and their babies die from causes which could be prevented with timely access to quality care. To understand the circumstances around these deaths and use such insights to guide prevention efforts, health authorities use a process called Maternal and Perinatal Death Surveillance and Response (MPDSR). This process involves identifying, reviewing, and learning from every maternal and newborn death to improve care and prevent similar future events. In this study, we examined the evolution of MPDSR policies and practices in Nigeria between 2003 and 2025. By reviewing government documents and studies, we identified four key stages of development: the early introduction of hospital-based death reviews, the inclusion of community deaths, the expansion to include perinatal and child deaths, and recent community-based models led by state governments. Despite these advancements, MPDSR implementation is inconsistent across the country. Progress is slowed by state differences in technical skill, political will and over-reliance on external donor funding. In addition, a major barrier is the absence of a national law, meaning states are not legally required to adopt or sustain MPDSR. However, states like Lagos and Kaduna are leading the way by developing successful community systems that link data from health facilities and local communities. The study concludes that to make MPDSR fully operational nationwide, Nigeria needs stronger national coordination, a reliable source of funding, and robust legal frameworks. Strengthening these systems is vital for saving lives of women and newborns and ensuring greater accountability within the Nigerian health system.

**Supplementary Information:**

The online version contains supplementary material available at 10.1186/s12978-025-02196-7.

## Background

Maternal and perinatal deaths remain major public health concerns globally. In 2023, an estimated 260,000 maternal deaths and 1.9 million stillbirths occurred, the majority preventable with timely and appropriate care [1, 2]. Over 98% of these deaths were in low- and middle-income countries (LMICs), with Nigeria contributing the highest number of maternal deaths (75,000) and the third highest number of stillbirths (184,000) worldwide in 2023 [1, 2]. The leading causes of maternal and perinatal mortalities have remained the same in the past two decades [1, 2].

In Nigeria, weak civil registration and vital statistics (CRVS) systems result in many maternal and perinatal deaths going undocumented, especially those occurring outside health facilities [3], which account for 59% of childbirths [4]. Understanding the circumstances around these deaths through death reviews is essential to guide prevention efforts [5]. Narratives from women, their families and other community members provide valuable insights into contextual and systemic factors not contributory captured in clinical records. Such accounts are vital for a comprehensive understanding of the events leading to death and often illuminate key elements of the ‘three delays’ framework, including delays in recognising danger signs, deciding to seek care, reaching appropriate facilities, and receiving timely and adequate care [6].

Structured maternal death reviews began globally with the United Kingdom’s Confidential Enquiry into Maternal Deaths in 1952, which paved the way for a formal process of maternal death reviews (MDR), enabling learning from avoidable maternal deaths [7]. With the very high burden of maternal mortality in LMICs, the World Health Organization (WHO) encouraged and supported health system leaders in LMICs to prioritise death reviews as a strategy for reducing maternal deaths [8]. This approach was further amplified during the launch of the Safe Motherhood Initiative at the International Safe Motherhood Conference in Nairobi, Kenya, in February 1987 [8]. During this conference, convened by the World Bank, WHO and United Nations Population Fund (UNFPA), there was a call to reduce maternal mortality in developing countries by 50% in a decade [9, 10]. To support countries in achieving this goal, the WHO led a range of initiatives throughout the 1990s, including the development of technical guidance for improving the quality of maternal health services [11], promotion of confidential enquiries and facility-based audits, and the integration of death reviews into national maternal health programmes [12]. These efforts were reinforced by advocacy campaigns, key publications [13], and the influential documentary ‘*Why Did Mrs X Die?*’, inspired by Professor Mahmoud Fathalla’s “road to maternal death” framework [14]. The International Conference on Population and Development in Cairo in 1994 further strengthened global commitments by placing reproductive health and rights at the centre of development goals, especially for high-burden countries [15].

Facility-based death reviews, which were common between the 1990 s and the early 2000 s, provided important insights into the quality of care but often overlook broader contextual factors beyond the health system [16, 17]. A landmark development in global efforts to scale implementation of MDR occurred in 2004 when the WHO released the technical guideline *“Beyond the Numbers: Reviewing Maternal Deaths and Complications to Make Pregnancy Safer”* [18]. Through this guideline, the WHO provided comprehensive guidelines for systematically reviewing maternal deaths and severe morbidities, regardless of where they occurred, and urged all countries to establish a maternal death and complications audit system to improve pregnancy outcomes [18].Subsequently, the WHO conceptualised the Maternal and Perinatal Death Surveillance and Response (MPDSR) system as a systematic way of capturing how many women died, where they died and why they died. MPDSR aims to reduce preventable deaths by identifying, reporting and reviewing maternal and perinatal deaths wherever they occur to inform actions aimed at improving the quality of care [5, 19]. Verbal and social autopsies, though limited in diagnostic precision, are recommended to complement facility-based reviews by revealing contributing factors such as delays in seeking care and broader social determinants. However, MPDSR implementation has varied widely across countries, with most systems focused primarily on facility-based data [5]. A previous article mapped implementation in Kenya and reported lessons learnt [20]. However, no such article has been published in Nigeria, despite the country’s high burden of maternal deaths and stillbirths and the criticality of MPDSR in helping to understand and address the problem. In this article, we present a historical timeline and policy analysis of the implementation of MPDSR in Nigeria.

## Methods

### Study design

This historical timeline and policy analysis based on review of published documents adopted principles and procedures of systematic review methods to retrieve relevant articles [21]. The historical timeline analysis involved reviewing the retrieved articles and extracting relevant data to create a chronological order of events and dates to understand the evolution of MPDSR implementation in Nigeria. This approach allows for visualising and understanding the sequence of events, identifying patterns, and recognising broad historical trends. For the policy analysis, we drew on the Walt and Gilson policy triangle framework [22] to critically examine the content, context, process, and actors influencing implementation and effectiveness, while reflecting on the implications of these changes for equity and sustainability.

### Search strategy

We systematically searched electronic bibliographic databases (PubMed, Scopus, Directory of Open Access Journal and Google Scholar) for relevant articles between February 21 and March 14, 2025 (Supplementary file 1). For the literature search, a combination of different keywords was adapted to suit each database to identify relevant articles. We did not apply any date or language restrictions to maximise the scope of our article retrieval. Recognising the relevance of grey literature to our study, a search of grey literature sources was conducted to retrieve relevant documents. While no gold standard exists for grey literature searching, this study drew on the strategy used by Godin and colleagues [23], with the aim of ensuring that the search methods used were explicit, reproducible and identified all relevant documents. We searched grey literature databases such as Open Grey and Grey Literature Report, as well as websites of organisations such as WHO, UNFPA, Federal and State Ministries of Health in Nigeria.

Search terms used for both peer-reviewed and grey literature search broadly included words and synonyms within two groups: (1) MPDSR, MDR, audit and (2) Nigeria (and all the 36 states including the Federal Capital Territory). We complemented or search results by hand-searching the reference lists in the relevant retrieved documents and engaging key stakeholders in the Ministry of Health and the Society of Gynaecology and Obstetrics of Nigeria (SOGON), a professional body of obstetricians and gynaecologists with a presence in all Nigerian states which has played a pivotal role in supporting and advocating for maternal death reviews in the country.

### Screening

Three authors (FMA, OIA and UG-A) conducted the initial screening of the title and abstract/executive summary/overview of retrieved documents. We entered the titles and respective weblinks of potentially relevant documents into Microsoft Excel and the document stored in Mendeley Desktop. Two authors (FMA and UG-A) independently assessed the stored full texts of documents for inclusion using the predefined inclusion criterion of document describing at least an event or role of an actor that is part of MPDSR implementation in Nigeria. Disagreements about eligibility were resolved by consensus and where necessary by discussing with the senior author (AB-T). Documents were excluded if it was difficult to extricate specific MPDSR-implementation related events relating to Nigeria from a wider pool of countries.

### Data extraction and analysis

We extracted relevant information using a pre-developed data extraction sheet. UG-A conducted the initial data extraction and independently cross-checked by FMA, OIA, DI, and AB-T to ensure consistency and accuracy. Extracted data included the title, author(s), year of publication, stated objectives of the document, level of reporting (national or state), MPDSR type (facility-based, community-based, or combined), key events with corresponding dates, and thematic categories informed by Walt and Gilson’s health policy triangle framework [22]. Events were coded with their associated dates, and categories were generated iteratively to identify broad patterns over defined time periods within the review timeline. These events were then plotted and visually presented to illustrate their chronological evolution. The health policy triangle framework [22] was subsequently used to guide our appraisal of MPDSR implementation in Nigeria with findings presented according to the components of the framework: context, content, actors, and process.

## Results and discussion

**Fig. 1:**
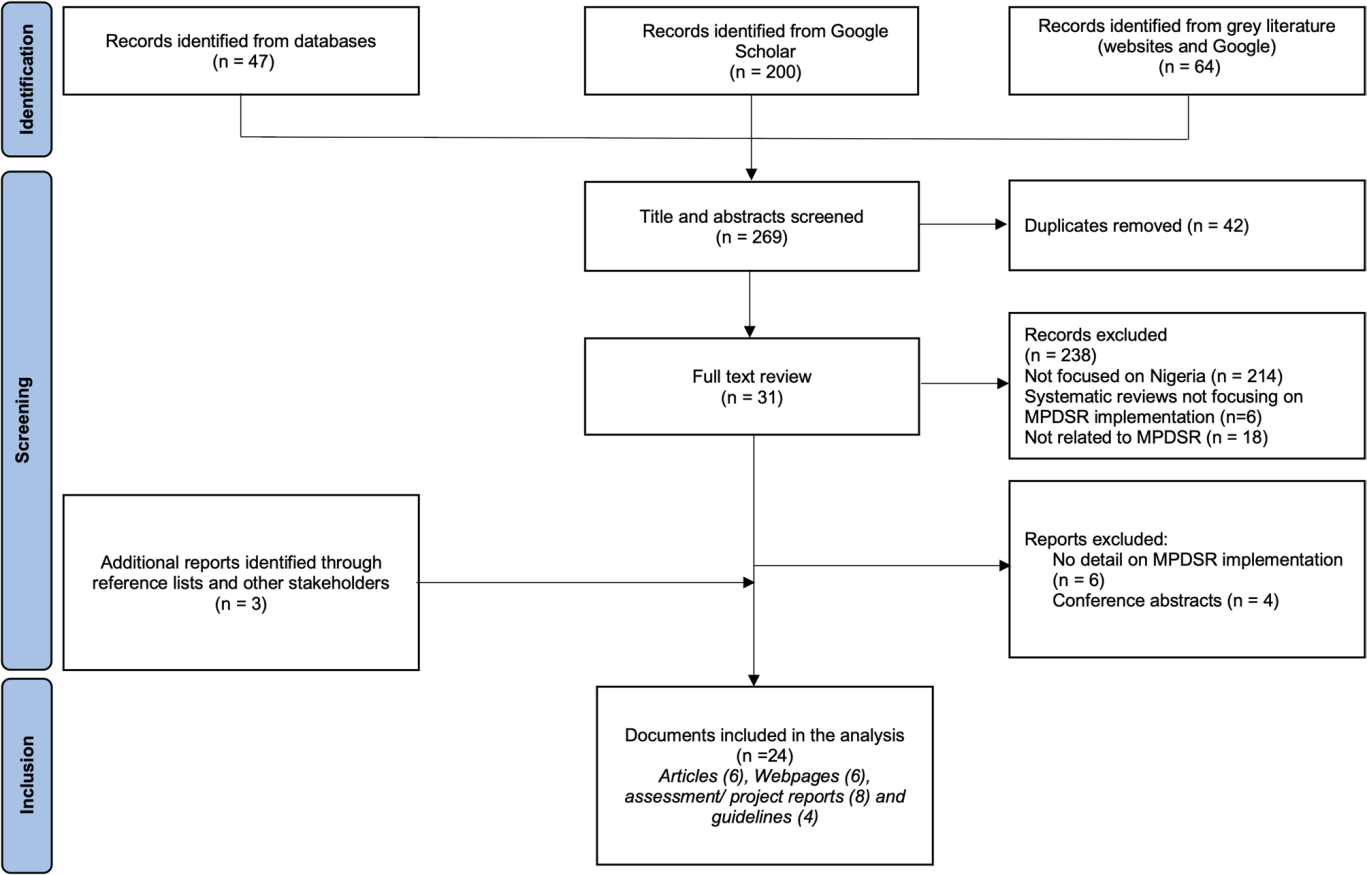
PRISMA diagram showing search results

We retrieved 311 records from all searches. Following deduplication, title and abstract screening, 31 documents were retained for full text review. Of these, 10 were excluded (six due to lack of detail on MPDSR implementation and four being conference abstracts). An additional three documents were identified through reference lists and stakeholder engagement. In total, 24 documents were included in the final analysis [5, 16, 24–45] [Figure 1]. Table 1 provides additional information on the author names, year of publication, title, and type of documents. Eight were reports [5, 27, 31, 34–36, 41, 44], six were research articles [16, 24–26, 28, 37], six were websites [29, 30, 38, 39, 42, 43], and four were guidelines [32, 33, 40, 45].

**Table 1:** Type of documents retrieved for analysis

S/*N*	Authors and year	Title	Document type
1	Hussein et al., 2016 [16]	Maternal death and obstetric care audits in Nigeria: a systematic review of barriers and enabling factors in the provision of emergency care	Research article
2	Hussein and Okonofua, 2012 [24]	Time for action: audit, accountability and confidential enquiries into maternal deaths in Nigeria	Research article
3	Pearson, deBernis, and Shoo, 2009 [25]	Maternal death review in Africa	Research article
4	Achem and Agboghoroma, 2014 [26]	Setting up facility-based maternal death reviews in Nigeria.	Research article
5	Okaro, J.M. and Iyoke, C.A. (2010) [28]	The Society of Gynaecology and Obstetrics of Nigeria (SOGON) plan for sustainable reduction in maternal mortality: a review	Research article
6	Tilton et al., 2023 [37]	Implementing Community-Maternal and Perinatal Death Surveillance and Response to identify and prevent maternal and perinatal mortality in Kaduna State, Nigeria: Results and lessons from a pilot study.	Research article
7	Federal Ministry of Health, 2015 [45]	National Guidelines for Maternal and Perinatal Death Surveillance and Response in Nigeria.	Guideline
8	World Health Organization, 2021 [32]	Maternal and perinatal death surveillance and response: materials to support implementation.	Guideline
9	World Health Organization, 2016 [33]	Making Every Baby Count: Audit and review of stillbirths and neonatal deaths, Highlights from the World Health Organization.	Guideline
10	Lagos State Primary Healthcare Board and Project Aisha, 2024 [40]	Community MPCDSR in Lagos State: Implementation Guidelines.	Guideline
11	World Health Organization, 2016 [27]	Time to respond: A report on the global implementation of Maternal Death Surveillance and Response.	Report
12	Okonta, Tetsola, and Obiazor, 2019 [31]	Delta State Maternal and Perinatal Death Surveillance and Response Report.	Report
13	World Health Organization, 2024 [5]	Maternal and perinatal death surveillance and response: global report on decade of implementation.	Report
14	Society of Gynaecology and Obstetrics of Nigeria, 2018 [44]	Annual Report of Maternal Death Review Project	Report
15	Maternal and Child Survival Program, 2017 [41]	Assessment of Maternal and Perinatal Death Surveillance and Response Implementation in Ebonyi and Kogi States, Nigeria.	Report
16	Options, Kaduna State Primary Healthcare Board and Evidence 4 Action (Mamaye), 2022 [34]	c-MPDSR Endline Report	Report
17	Africare, Nigeria Health Watch and EpiAfric, 2020 [35]	Why are women dying while giving birth In Nigeria?	Report
18	Oladapo, Kinney, and Save the Children, 2017 [36]	Assessment of Maternal and Perinatal Death Surveillance and Response Implementation in Nigeria.	Report
19	Lagos State Verbal & Social Autopsy - Sample Registration System, 2025 [38]	About Lagos State Verbal & Social Autopsy - Sample Registration System (LVASA-SRS)	Webpage
20	African Centre of Excellence for Population Health and Policy, 2025 [39]	Kano State Surveillance for Evidence and Policy	Webpage
21	World Health Organization, 2021 [42]	WHO collaborates with Ministry of Health to tackle maternal and perinatal mortality.	Webpage
22	Onyedinefu, 2020 [43]	FG launches verbal, autopsy report to tackle infant, maternal mortality	Webpage
23	Obi and Sumain, 2015 [29]	Closing the obstetrics gap	Webpage
24	MacArthur Foundation, 2025 [30]	Society of Gynaecology and Obstetrics of Nigeria	Webpage

CHAI: Clinton Health Access Initiative; FCT: Federal Capital territory; FMoH – Federal Ministry of Health; HMOH: Honourable Minister of Health; MDR: Maternal Death Review: NISONM: Nigerian Society of Neonatal Medicine: NPHCDA: National Primary Health Care Development Agency; SNL: Saving Newborn Lives; SOGON: Society of Gynaecology and Obstetrics of Nigeria; UNFPA: United Nations Population Fund; UNICEF: United Nations Children’s Fund; WHO: World Health Organization

### Evolution of MPDSR implementation in Nigeria

**Fig. 2:**
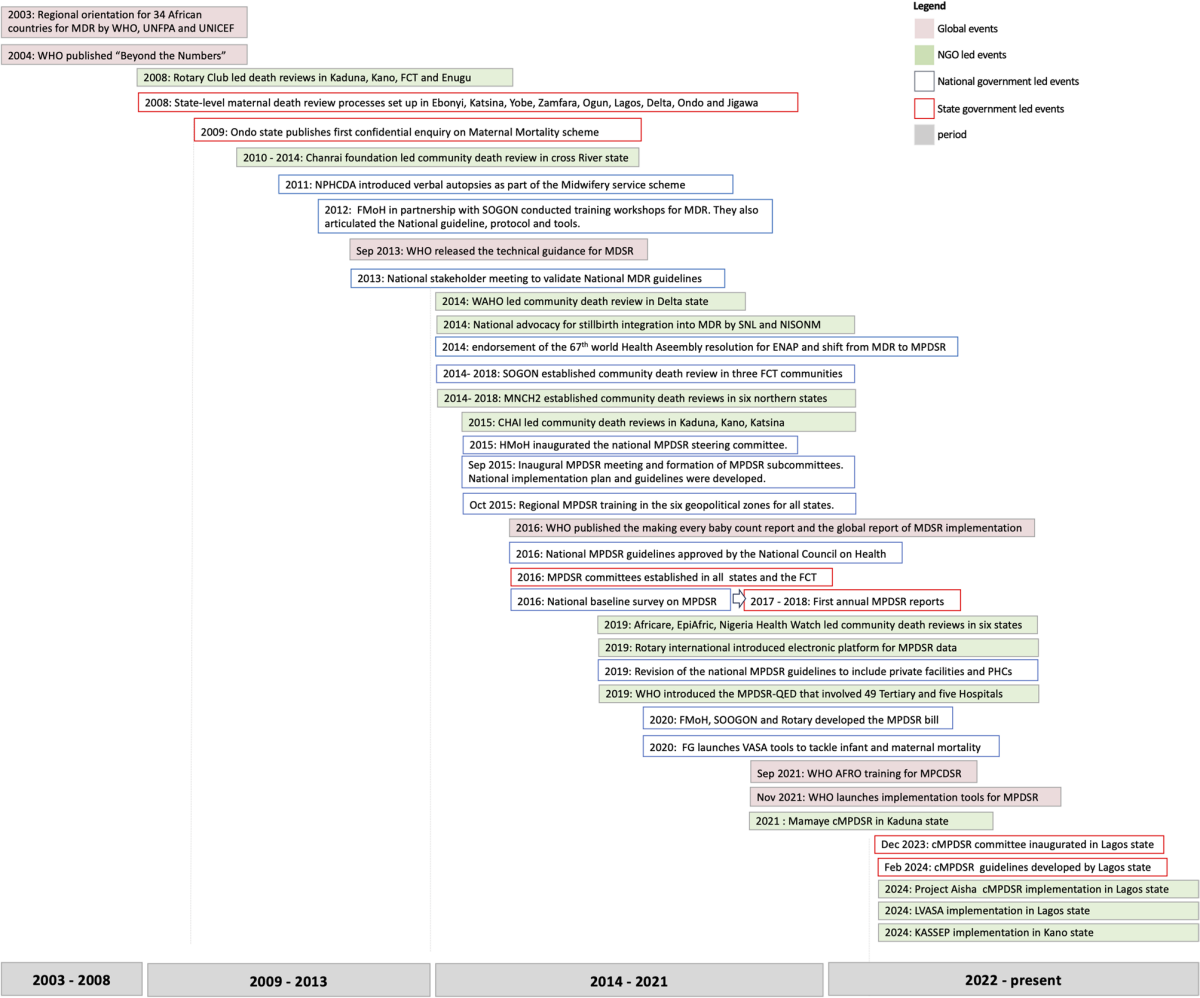
Evolution of MPDSR in Nigeria

Following our mapping of events related to MPDSR implementation in Nigeria, we identified four key phases: (1) Setting up facility-based maternal death and morbidity reviews (2003–2008), (2) Extending maternal death reviews into the community (2009–2013), (3) Adding the ‘P’ and the ‘C’ to MDSR while making efforts to institutionalise and strengthen systems (2014–2021), and (4) Deemphasising the ‘C’ for child and emphasising another ‘C’ in piloting ‘c’ommunity-based MPDSR (2022–Present). Details of the events during these phases are presented below and visualised in Fig. 2.

#### Setting up facility-based maternal death and morbidity reviews (2003–2008)

Although maternal death and morbidity reviews had long been conducted in Nigerian hospitals, particularly in tertiary centres as part of clinical training for health workers, these early efforts were often unstructured, lacked standardised protocols, and varied widely in quality and approach [16, 24, 45]. Critically, they failed to capture the majority of the maternal deaths which occurred in the communities, leading to significant gaps in surveillance. Early review activities remained largely hospital-focused, falling short of implementing global guidance that emphasised the importance of community-level reviews in high-burden settings [18, 36].

In 2003, Nigeria participated in a regional initiative led by WHO, UNFPA, and United Nations Children’s Fund aimed at institutionalising MDR across African countries. As part of this effort, national MDR committees and programme managers from 34 African countries, including Nigeria, attended regional orientation workshops where countries were introduced to five MDR methodologies: verbal autopsy, facility-based MDR, near-miss reviews, confidential enquiries, and criterion-based clinical audits [25]. At this orientation workshop, Nigeria, alongside other participating countries, developed a provisional national MDR plan. The Nigerian government attempted to establish a national-level MDR system following this orientation, but the effort was ultimately unsuccessful [25, 26]. This failure was attributed to weak government ownership, inadequate resources, legal and policy gaps, poor staff training, limited stakeholder engagement, and a lack of action on findings [26]. The governance of MDR in Nigeria has predominantly followed a top-down approach, with policies and guidelines developed at the federal level and expected to cascade down to the state and local government levels [27, 36]. However, adoption at the subnational levels has been hindered by disparities in resource availability, weak enforcement mechanisms and inadequate health governance in the country, leading to inconsistencies in maternal and perinatal death surveillance and response across the country [36]. By leveraging its technical expertise and local presence, SOGON has contributed to advancing the quality and uptake of MDR practices where government efforts have fallen short [28]. Separately, the release of the 2004 WHO report further reinforced the global and regional importance of structured review systems and indirectly shaped Nigeria’s evolving response in this domain.

In 2008, the Rotarian Action Group for Population and Development, particularly the Rotary Clubs in Nigeria, Austria, and Germany, collaborated with the state governments of Kano, Kaduna, Enugu and the Federal Capital Territory (FCT) Abuja, to establish the Obstetrics Quality Assurance system (OQA) in selected hospitals [29]. The key mandate of the OQA was to review maternal and perinatal deaths in the health facilities, identify and resolve preventable causes, and engage with communities to strengthen local prevention efforts [29]. Around the same period, with support from state and international donors, other states such as Ebonyi, Katsina, Yobe, Zamfara, Ogun, Lagos, Delta, Ondo, and Jigawa began implementing their own maternal death review processes [5, 45]. These death review processes were primarily hospital-focused, and while the OQA project actively engaged local communities for prevention.

#### Extending maternal death reviews into the community (2009–2013)

One of the earliest efforts to expand maternal death reviews beyond health facilities and formally include community deaths was the 2009 Ondo State Confidential Enquiry into Maternal Mortality [36]. This scheme was initiated by the then state governor, who was also a physician, the initiative had a strong political backing. To support its implementation, the state government enacted a law to mandate prompt reporting of maternal deaths occurring both in hospitals and in communities. The Confidential Enquiry Committee was responsible for systematically reviewing the deaths and making evidence-based recommendations to inform policy and actions [36]. In 2010, the Chanrai Foundation, a non-governmental organisation, in collaboration with the Cross Rivers State government, introduced community-level maternal death reviews in the State using verbal autopsies [36]. This initiative was continued by the Department of Primary Health Care of the Cross River State Primary Health Care Development Agency until 2014 [36].

The first large-scale implementation of community-level maternal death review using verbal autopsies in Nigeria was carried out by the National Primary Health Care Development Agency (NPHCDA) through the Midwives Service Scheme (MSS), which empowered midwives to conduct these reviews [36, 46]. This was implemented from 2011 to 2013 in 19 states in Nigeria, and the FCT. During this period, efforts to develop national guidelines for MDR continued and were primarily sustained by high-level stakeholder engagement. Key developments included a three-day MDR training workshop in March 2012 in Abuja, organised by the Federal Ministry of Health in partnership with the SOGON and the International Federation of Gynecology and Obstetrics. This was followed by an additional one-day workshop on April 12, 2012, and a two-day workshop on July 4–5, 2012, to draft the national guidelines, protocols and tools for conducting MDR in Nigeria [46].

The release of the WHO technical guidance for Maternal Death Surveillance and Response (MDSR) in September 2013 coincided with ongoing efforts in Nigeria to develop national guidelines [19]. Like the *Beyond the numbers technical guideline*, the technical guidance for MDSR underscored the importance of community-level death reviews in high-burden settings using verbal autopsies and encouraged joint reviews involving community representatives to better understand the social and systemic contributors to maternal deaths. Recognising the importance of understanding the causes of maternal deaths to inform effective interventions, SOGON, with support from the MacArthur Foundation in 2013, piloted a four-year MDR project in three states and led nationwide advocacy for its adoption [30, 44]. Subsequently, a national stakeholder meeting was convened later in 2013 by the Federal Ministry of Health (FMoH) to review and validate the zero draft of the Nigeria death review guidelines [45]. While this guideline contained guidance for community death reviews using verbal autopsies, the initial rollout plan focused on piloting death reviews in all tertiary facilities in the country, in addition to the ongoing efforts in Lagos, Ogun, Delta and the FCT, where SOGON was already leading MDR implementation pilots [46].

#### Adding the ‘P’ and the ‘C’ to MDSR while making efforts to institutionalise and strengthen systems (2014–2021)

The SOGON pilot in the FCT in 2014 included verbal autopsy in Kogo, Kwali and Gbagalape communities in Bwari, Lambata and Abuja municipal area councils, respectively [36]. The same year, the West African Health Organization in partnership with the Delta State Ministry of Health and Delta State Primary Health Care Development Agency launched a pilot community-based death review project in one local government area [31, 36] This initiative laid the foundation for the subsequent establishment of the health facility and state-level MDR processes in Delta State in partnership with SOGON [36]. These localised pilots reflected a growing commitment to expand maternal death surveillance in Nigeria.

Efforts to institutionalise MDR gained national momentum following the development of national guidelines for MDSR. For example, the Nigeria Maternal, Neonatal and Child Health Programme, a country-led, UK government-funded initiative, launched a five-year MDR project across six states: Kaduna, Kano, Katsina, Jigawa, Yobe, and Zamfara in 2014 [36]. This project included both facility-level MDR and community verbal autopsies, further embedding MDR into routine health governance structures. Also in 2014, the Saving Newborn Lives project of Save the Children, in collaboration with the Nigerian Society of Neonatal Medicine, led advocacy for the integration of stillbirths and neonatal deaths into the existing MDSR system [41]. Their technical support enabled the FMoH to revise MDSR tools and guidelines to include perinatal death reviews. This aligned with Nigeria’s endorsement of the 67th World Health Assembly resolution on the ‘*Every Newborn Action Plan’* and supported a transition toward the broader MPDSR framework [46]. SOGON and other stakeholders reinforced this shift through sustained advocacy. In 2015, Clinton Health Access Initiative implemented an MDR project in health facilities and surrounding communities using verbal autopsies in Kaduna, Kano and Katsina states [36].

A key step toward institutionalising MPDSR in Nigeria occurred in March 2015 with the inauguration of the National MPDSR Steering Committee [46]. At its inaugural meeting in September 2015, subcommittees were formed to oversee various components of MPDSR implementation [46]. The national MPDSR implementation plan was also developed at this meeting and later submitted for approval to the Permanent Secretary for Health. By the end of 2015, Nigeria’s finalised MPDSR guidelines and implementation plan were made available, offering a national framework for the systematic review of maternal and perinatal deaths, including those occurring at the community level [45]. To facilitate the subnational operationalisation of MPDSR, a nationwide training of states steering committee members was conducted between October and November 2015 across the six geopolitical zones in Nigeria, with participants tasked to champion the establishment and advancing MPDSR efforts within their respective states [46].

Progress continued in 2016 with the release of two key global reports: *Making Every Baby Count* [33] and *Time to Respond: A Report on the Global Implementation of Maternal Death Surveillance and Response (MDSR)* [27], both of which emphasised the critical role of comprehensive audits of hospital and community-level maternal and perinatal deaths to improve maternal and newborn survival. At the national level, the national MPDSR guidelines were officially approved by the National Council of Health, paving the way for widespread adoption and enabling states to adopt and contextualise them to their setting. Building on the achievements of their initial MDR project, SOGON expanded its efforts in six facilities in Abuja in 2016, aligning with the national MPDSR guidelines by incorporating a perinatal component into the review process, with additional funding from MacArthur Foundation [30]. To track progress, a national baseline assessment of MPDSR implementation was conducted in 2016, informing subsequent activities and culminating in the production of Nigeria’s first MPDSR annual reports in 2017 and 2018 [46].

As attention increasingly shifted toward strengthening community-level surveillance, several new initiatives emerged from 2019 onward. For instance, the Giving Birth in Nigeria project, an 18-month community-based MDR intervention, was launched in January 2019 in Bauchi, Bayelsa, Ebonyi, Kebbi, Lagos, Niger, and the FCT [35]. Funded by MSD for Mothers, the project focused specifically on improving community accountability and response to maternal deaths. Meanwhile, at the facility level, Rotary International partnered with the FMoH to introduce the *National Obstetric Quality Assurance (NOQA)* electronic platform [46]. This system was designed to support MPDSR implementation in health facilities by centralising data management, thereby improving data quality, surveillance, and accountability.

Recognising the gap in surveillance in private sector facilities, primary healthcare centres and the community level, the FMoH led the revision of the National MPDSR Guidelines and Training Toolkits, resulting in the second edition of the national guidelines in 2019 [46]. Despite these advancements, the uptake of MPDSR in communities remained minimal, with progress concentrated mainly in hospitals. In the same year, WHO introduced the two-year Maternal and Perinatal Database for Quality, Equity and Dignity (*MPD-4-QED) initiative*, which integrated quality improvement strategies with routine MPDSR reviews [42, 46]. This MSD for Mothers funded initiative focused on strengthening quality improvement through routine MPDSR reviews in 49 tertiary hospitals and five additional health facilities, with the ambitious goal of halving maternal deaths in Nigeria [42].

In 2020, Nigeria made an attempt to legally institutionalise MPDSR through the development of an MPDSR bill [46]. This was led by the FMoH with support from the Chairman of the Senate Committee on Health, SOGON and Rotary International. The bill aimed to consolidate the various strands of MPDSR implementation, including national efforts by the FMoH, community-level processes led by the NPHCDA, and hospital-based reviews under MPD-4-QED. The bill sought to provide a unified, standardised, and legally backed framework for capturing, reviewing, and responding to maternal and perinatal deaths across all levels of the health system. While some states such as Kogi and Ebonyi have taken proactive steps to institutionalise MPDSR through state-level legislation, the absence of a national legal framework limits harmonised implementation and undermines accountability mechanisms across the country [5, 47]. Later in 2020, the Federal Government, in collaboration with the National Population Commission, launched the Verbal and Social Autopsy tool to investigate causes of under-five mortality between 2013 and 2018 [43]. While focused more broadly on child mortality, this initiative reflected a broader integration of death surveillance into Nigeria’s health information system.

Integration continued into 2021, and on September 9, 2021, the WHO Regional Office for Africa Office organised a pan-African virtual training to guide member countries on adopting and integrating the new Child Death Audit into existing death review mechanisms. In response, Nigeria developed the *Maternal*,* Perinatal*,* and Child Death Surveillance and Response (MPCDSR) Guidelines* to incorporate child death audits into the existing MPDSR framework [46]. The document included comprehensive guidance on conducting community verbal autopsies. However, the integration of child death reviews into the MPDSR system in Nigeria remains limited, with little evidence of the systematic inclusion of child mortality data within the existing framework. On the global level, the WHO published the *Implementation Tools for Maternal and Perinatal Death Surveillance and Response*, which included guidance on conducting both facility-based reviews and community verbal autopsies in high-burden settings on November 11, 2021 [32]. These tools provide standardised methodologies for the holistic conduct of MPDSR.

#### Deemphasising the ‘C’ for child and emphasising another ‘C’ in piloting ‘c’ommunity-based MPDSR (2022–Present)

Despite the child death component of MPCDSR remaining underdeveloped, recent efforts have shifted toward piloting community-based MPDSR. Implementation of community-based MPDSR in Nigeria has accelerated since 2022, driven by international donor funding and the initiative of a few proactive states. In Kaduna State, for example, E4A-MamaYe and Population and Reproductive Health Initiative partnered with the Kaduna State Primary Healthcare Board to co-design and pilot a community-based MPDSR project between December 2021 and July 2022 in one LGA [34, 37]. The implementation process required state MPDSR actors to adapt the national guidelines and existing community-based MPDSR models to the Kaduna State context [34]. Key decisions included leveraging the capacities of existing ward development committee members and PHC staff rather than hiring new staff and localising community-based MPDSR governance at the LGA level. They subsequently mapped out and extensively engaged relevant community stakeholders which ensured effective implementation and sustainability [34].

In a similar vein, in 2023, Lagos State adapted the national guidelines and began a pilot community-based MPDSR project through a collaboration between the Lagos State Primary Health Care Board (LSPHCB), UNFPA, Health Strategy and Delivery Foundation, mDoc, Ingress Health Partners, and the Institute for Healthcare Improvement [40]. This project dubbed, ‘Project Aisha’, a three-year MSD-funded quality improvement initiative which involved strengthening community-level accountability for maternal and newborn deaths as a core focus. On September 6, 2023, Lagos State convened a one-day workshop with key stakeholders to review the national guidelines and existing implementation models for community-based MPDSR. The Kaduna State community-based MPDSR implementation model was favoured, prompting an extensive virtual knowledge exchange meeting between the Kaduna State Primary Health Care Board community-based MPDSR team and the LSPHCDA on September 26, 2023. Insights from the meeting informed a final validation meeting on October 6, 2023, which now included key community stakeholders from the proposed pilot LGA (Ifako Ijaye) to ensure their inputs and perspectives were integrated. Subsequently, the LSPHCB formally inaugurated its community-based MPDSR subcommittee to oversee community-level maternal and perinatal death surveillance in Lagos State. By February 2024, the state finalised its community-based MPDSR guidelines making way for implementation [40].

In parallel, the Kano and Lagos state governments, with support from the Bill and Melinda Gates Foundation, initiated verbal and social autopsy projects in 2024, aiming to generate population-level estimates of maternal deaths and stillbirths while identifying underlying causes. In Lagos state, the Lagos Verbal Autopsy and Social Autopsy - Sample Registration System is led by the state government in collaboration with researchers from the Lagos University Teaching Hospital, Lagos State University Teaching Hospital, and the London School of Hygiene & Tropical Medicine [38]. The project commenced in April 2024 across all LGAs in Lagos and involves collecting data on pregnancies in the preceding 12 months, enumerating currently pregnant women, and following them through delivery. All maternal deaths and stillbirths within the sample enumeration areas will be reviewed using verbal and social autopsy methods [38]. Similarly, the Kano State Surveillance for Evidence and Policy project follows a similar implementation framework. The project commenced in 2024 and is being implemented by researchers at Bayero University, Kano and Yusuf Maitama Sule University, with the aim of generating actionable evidence to inform maternal and perinatal health policies and interventions at the state level [39].

### Appraising the implementation of MPDSR in Nigeria

Drawing on the Walt and Gilson policy triangle framework [22], this appraisal interrogates the policy content, context, processes, and actor dynamics shaping the implementation and effectiveness of Nigeria’s MPDSR implementation.

#### MPDSR policy content

Nigeria’s national MPDSR policy and guidelines reflect global best practices in framing surveillance as a cycle of identification, notification, review, and response [5, 36]. Initially released in 2015 and revised in 2019 and 2021, the national guidelines have broadened in scope to include perinatal and child deaths. However, implementation has not kept pace with these expansions, resulting in a fragmented, under-resourced, and inconsistent landscape across states and facility types [36, 48].The 2015 national rollout, based on earlier facility-based pilots in Lagos, FCT, Delta, and Ondo, aimed to integrate MPDSR into routine health systems nationwide. Yet, by 2017, a national baseline assessment revealed persistent shortcomings: over half of health facilities lacked trained personnel or clear reporting mechanisms, and only a third routinely conducted maternal death reviews. Stillbirth reviews were even less frequent, hindered by weak classification systems and inadequate root cause analysis [36]. These challenges reflect a broader disconnect between national policy and on-the-ground implementation, shaped by variations in state context and capacity.

The expansion in scope from Maternal Death Review (MDR) to Maternal and Perinatal Death Surveillance and Response (MPDSR), and more recently to Maternal, Perinatal and Child Death Surveillance and Response (MPCDSR) in Nigeria, reflects a shift in scope and ambition toward comprehensive mortality surveillance. However, this expansion has occurred without the full rollout or institutionalisation of any single component. Although initially conceptualised as a unified programme, MPDSR has, in practice, split into separate facility- and community-based tracks. This unintended bifurcation reflects the kind of design-to-implementation disconnect described by Eboreime et al. (2021), where the coherence of health interventions is compromised during operationalisation, leading to what is effectively an implementation failure. Presently, facility-based MPDSR is relatively more established, with national tools, reporting structures, and some degree of routine implementation, while community-based components remain fragmented, under-integrated, and often externally driven. Even in states where it is functional, community-based MPDSR is often not well embedded within government-led health information systems [36, 48].

NPHCDA’s early effort to introduce community-level maternal death reviews using verbal autopsy through the Midwives Service Scheme (2011–2013) focused more on service delivery than surveillance [50]. Despite the inclusion of verbal autopsy in national MPDSR guidelines since 2015, meaningful community-based implementation only began through isolated, donor-supported pilots, such as in Ondo (2009), Cross River (2010), and Kaduna (2022). These implementation efforts have extended the reach of surveillance in the states, but the projects often operate in parallel to government systems, weakening alignment with national planning and accountability frameworks.

#### MPDSR policy context

The adoption of MPDSR in Nigeria aligns with a broader global agenda to institutionalise structured approaches to maternal and neonatal mortality reduction. MPDSR has been implemented in countries across diverse mortality contexts as a mechanism for health system strengthening. However, in Nigeria, the persistently high number of maternal and perinatal deaths which is now among the highest globally [1, 2], has created a particularly urgent impetus for implementation.

In addition to mortality statistics, the Nigerian context presents several structural and socio-political factors that influence MPDSR implementation. The country’s federal structure results in significant subnational variation in political will, institutional capacity, and resource availability. In the absence of a binding national legal framework, states bear responsibility for legislating and operationalising MPDSR, leading to uneven uptake. While some states, such as Ebonyi and Kogi, have passed dedicated MPDSR laws [5, 47], others have relied on policy guidelines or donor-supported protocols, often resulting in fragmented or symbolic MPDSR implementation. Despite the existence of comprehensive national MPDSR guidelines, effective implementation at the subnational level remains limited. Nigeria’s federal structure devolves health governance to states, which vary markedly in political will, institutional capacity, and resource allocation. In the absence of a binding national legal framework, national directives rely on voluntary state adoption, resulting in uneven uptake. Moreover, limited technical capacity, weak accountability mechanisms, and dependence on donor-driven initiatives hinder sustained institutionalisation. Consequently, policies formulated at the national level often do not cascade effectively to states, where implementation depends on local priorities, leadership, and fiscal space. Critically, the lack of legal backing also means that MPDSR participants are not indemnified against blame, professional censure, or litigation, factors which continue to undermine open, honest, and effective review processes. Also, the heavy donor involvement, accounting for over 90% of funding until recently [5], has reinforced a reliance on parallel systems, undermining national ownership and long-term sustainability. While some states, such as Lagos, Ekiti, Ondo, Delta, and the FCT, have begun to allocate budgetary resources to MPDSR, most states continue to depend on extra-budgetary or donor support, with hospital managers often financing review processes through internally generated revenue [5].

Another important context relates to the continued use of traditional birth attendants (TBAs), who are often deeply embedded within communities, for childbirth, particularly in rural areas where a substantial proportion of births occur outside health facilities. In Nigeria, TBAs typically operate outside the formal health system and are often not involved in the MPDSR process, which limits surveillance over outcomes of their service provision [47]. This lack of integration results in significant blind spots for mortality surveillance and undermines efforts to create a comprehensive national picture.

Even within the formal health system, there is a plurality of private providers, including faith-based, for-profit, informal clinics, and chemists, that operate independently of the public sector. Many are unregistered or lack routine oversight, making it difficult to enforce adherence to MPDSR protocols. Their sheer number and diversity make effective regulation and integration into national surveillance systems extremely difficult, undermining efforts to ensure data quality, accountability, and comprehensive death reporting.

Additionally, a pervasive blame culture within Nigerian society undermines the effectiveness of MPDSR implementation. In a context where accountability is often equated with punishment, mistakes are rarely met with constructive engagement. This societal tendency permeates health institutions, shaping the dynamics of MPDSR review committees. Within these forums, defensive posturing by clinicians is common, particularly in the presence of hierarchical structures that discourage open dialogue [36, 51]. As a result, reviews often shift from learning and improvement to fault-finding, limiting MPDSR’s potential as a system-strengthening tool.

#### Actors and interests

MPDSR processes operate within a complex landscape of actors, where power imbalances and competing priorities influence how implementation unfolds. In several LMICs, as we found in Nigeria, coordination, inclusivity, and the sustainability of response efforts have been challenged by political interference, donor-driven agendas [52], strained health worker–community relations, often shaped by blame or fear of legal repercussions [53] and the instability of crisis-affected settings [54].

At the national level in Nigeria, the FMoH, development agencies (e.g. WHO, UNFPA), and professional associations provide technical guidance and funding for MPDSR implementation. Importantly, the FMoH and the PHC Board often function as distinct actors, with the former driving facility-based MPDSR efforts through secondary and tertiary care platforms, while the PHC Board typically oversees community-level initiatives such as community-based MPDSR and social autopsies. The responsibility for operationalisation of MPDSR lies with subnational actors such as State Ministries of Health, hospital leadership, and community health committees, who often lack sufficient capacity or resources. Professional associations like SOGON have been instrumental in advancing MPDSR. Since the 2013–2015 MacArthur-supported pilot, SOGON has driven advocacy, training, and implementation support, filling gaps left by weak government leadership. Similarly, projects by Save the Children, the Clinton Health Access Initiative, and Rotary International helped pilot perinatal reviews and establish data platforms like NOQA.

Frontline health workers, who function as street-level bureaucrats, are central to implementing MPDSR and translating review findings into action. Across Nigeria, and more broadly in other LMICs, these actors often face significant barriers such as overwhelming workload, unclear role definitions, limited training, weak supervision, minimal feedback, and fear of blame [27, 37, 48, 55]. These challenges weaken the integrity and effectiveness of the surveillance cycle, particularly the ‘response’ component, leading to perfunctory implementation and undermining the transformative purpose of MPDSR [56].

Community actors, including traditional birth attendants, religious leaders, and local gatekeepers, play a vital role in improving the completeness of surveillance and facilitating local action [57]. Yet, their involvement often remains limited, driven more by project-based incentives than by sustained government engagement. Trust and collaboration between communities and health system actors are also critical to the success of such approaches. A recent study from Kenya highlighted deep-rooted scepticism among health workers about the competence and credibility of community members involved in MPDSR, which led to tokenistic engagement or their exclusion from meaningful participation in death reviews [56]. However, a similar study in Kenya showed that deliberate investments in building trust and clarifying community roles enabled stronger participation, improved death identification, and better uptake of recommendations [20]. In Nigeria, the 2022 community-based MPDSR pilot in Kaduna State offers a promising contrast. The study found that social autopsies were acceptable and valued by community members, leading to increased trust between community members and health facility staff [37]. This enhanced collaboration resulted in tangible actions, such as the closure of an unskilled maternity clinic, provision of 24-hour free transportation for pregnant women, and pre-emptive blood donations by community members. These outcomes suggest that deliberate efforts to build mutual respect, clarify roles, and strengthen collaborative mechanisms between communities and health system actors can enhance the credibility and effectiveness of community surveillance [58].

#### Process and implementation challenges

Our analysis reveals a persistent tension between top-down and bottom-up approaches. Nigeria’s MPDSR system has largely adopted a top-down model, with national guidelines disseminated to subnational levels with varying support [45]. While this ensures policy coherence, it frequently overlooks contextual realities, such as resource availability and community dynamics. In contrast, bottom-up initiatives such as state-led adaptations of community-based MPDSR are more contextually grounded but often lack the institutional support, financing, and political leverage required for national scale-up. Nigeria’s transition from MDR to MPDSR further illustrates these dynamics. States with stronger governance structures and dedicated support for MDR were better positioned to adopt MPDSR and embed it into routine practice [36]. Conversely, states with weaker health systems and limited resources struggled with uptake, resulting in fragmented implementation and limited institutionalisation. This variation underscores the critical role of subnational leadership, financing, and capacity in translating national policy into sustained action. Without this, surveillance risks becoming a performative exercise rather than a meaningful tool for learning and accountability.

In Nigeria, efforts to institutionalise community engagement in MPDSR have highlighted both the potential and challenges of embedding such mechanisms within routine health system processes. Evidence from other LMICs reinforces the value of institutionalised community engagement in MPDSR. In Malawi, community-led MPDSR processes supported by broad stakeholder involvement achieved high action completion rates: 82% at the community level, 67% in hospitals, and 65% in health centres, reflecting improved accountability and follow-through [59]. In Bangladesh, the Maternal and Neonatal Death Review system evolved into a national community-based MPDSR platform, with strong government commitment enabling the integration of death notification, review, and response into routine health system functions [5, 60]. South Africa’s approach also illustrates the importance of legal frameworks that mandate reporting and response actions, helping to institutionalise MPDSR as a routine and accountable health system function [20]. Together, these cases show that MPDSR mechanisms are most effective when embedded in government-led, system-wide structures that ensure sustainability, accountability, and responsiveness to local realities.

The response component of MPDSR which is the crucial step of translating review findings into corrective action remains critically weak in many Nigerian states. Despite efforts to institutionalise MPDSR through state-level legislation in some contexts, frontline health workers often fear blame or punitive consequences, which discourages open reporting and impedes implementation. Without strong legal protections and clear mechanisms for accountability, MPDSR frequently stalls at the review stage, with limited evidence of systematic follow-up, actionable planning, or monitoring of intervention uptake [36]. By contrast, examples from states such as Lagos, Jigawa, and Kano show that even modest review processes can trigger meaningful responses. In Jigawa, maternal death reviews led to the creation of blood donor clubs in response to haemorrhage-related deaths [61]. Similarly, in Kano, MDR findings were integrated into the State Medium Term Sector Strategy (2016–2018), leading to policy-level investments in blood banks and community health awareness [61]. These cases illustrate the potential of MPDSR to drive both local action and policy reform when the response component is actively prioritised.

### Strengths and limitations

This study is the first to systematically synthesise the historical and policy evolution of MPDSR implementation in Nigeria using the Walt and Gilson policy triangle. By examining actors, context, and processes, and drawing from both peer-reviewed and grey literature including government reports and programme evaluations the study offers a comprehensive and policy-relevant account. Methodological rigour was ensured through a systematic search, independent screening, and triangulation across document types. The study also critically assesses implementation depth, community engagement, legal institutionalisation, and donor dependence, contributing meaningfully to literature on health system governance in LMICs. However, despite multiple retrieval strategies and outreach to key informants, some relevant documents particularly unpublished or informal sources may have been missed. The historical timelines reflect the authors’ interpretations and may not fully capture undocumented or informal dynamics. Reliance on secondary data introduces potential limitations related to completeness, accuracy, and variable quality, especially in subnational reports. While the Walt and Gilson framework offers a strong policy lens, it does not explicitly engage with power dynamics or political economy in the period covered, which future research could address through more critical or decolonial approaches. Also, the static design of the framework limits its ability to capture the dynamic and temporal nature of policy evolution [62, 63]. We addressed this by applying the framework iteratively across defined time phases and triangulating evidence from multiple document types and stakeholder inputs to reflect changes over time.

### Pathway forward

To strengthen MPDSR nationally, Nigeria must adopt a hybrid model that merges central coordination with subnational autonomy and community participation. Although Nigeria already has a national MPDSR secretariat, its role should be strengthened to enhance state-level support through continuous capacity building, mobilising sustained funding, and improving performance monitoring with transparent reporting. It should also ensure that emerging community-based MPDSR efforts led by NPHCDA are effectively linked and coordinated with facility-based systems to prevent parallel structures from developing. Additionally, the secretariat should deepen collaboration with professional associations and communities and integrate MPDSR findings more effectively into health system planning. Ongoing legislative efforts to pass the MPDSR bill [25, 36] should be accelerated to provide legal backing for MPDSR, ensuring consistent implementation across states, safeguarding participant confidentiality, and institutionalising the practice beyond changing political administrations.

At the subnational level, states should be empowered and supported not only to integrate MPDSR into their annual health work plans and budgets but also to ensure consistent, quality implementation through dedicated funding, capacity building, and robust monitoring systems that translate plans into tangible action. In Kenya, counties were supported to implement MPDSR through national policy guidance, technical assistance, and financial backing, enabling subnational structures to adapt the tools to their contexts and progressively take ownership of the process [64, 65]. In Nigeria, states with stronger governance structures and dedicated MPDSR resources, such as Lagos State, have demonstrated more routine practice [36]. Expanding dedicated budget lines, supportive supervision, and state-level coordination mechanisms will help address the current unevenness in MPDSR implementation.

Data quality and use require strengthening. In Kenya, MPDSR data were integrated into the DHIS2 platform to support standardised data collection and promote use of findings for action, although evidence on improved responsiveness remains limited [20, 66]. Nigeria should fast-track the inclusion of MPDSR indicators into DHIS2, supported by investments in digital infrastructure, training for data managers, and user-friendly dashboards for decision-makers. High-quality data from MPDSR must not only be collected but also analysed and translated into action through local review meetings and follow-up mechanisms.

Community-based MPDSR must also be institutionalised through policy, not projects. As shown in the Kaduna pilot, community engagement through structured verbal and social autopsies, inclusive dialogue forums, and joint planning with health workers increased trust and reporting completeness [37, 67]. However, these successes remain isolated. Mechanisms for community death identification and notification should be standardised and linked to facility processes [67]. Engagement of male partners, traditional leaders, and women’s groups can enhance both accountability and local ownership of solutions [53, 58].

Finally, MPDSR should be reframed not merely as a surveillance tool but as a driver of equity-oriented, responsive health system transformation. Bandali et al. (2019) emphasise that MPDSR’s real power lies in closing the loop from review to action [65]. Without parallel investments in emergency transport, referral networks, and respectful maternity care, even the most robust MPDSR systems will struggle to achieve meaningful change (62). Nigeria must commit to translating MPDSR findings into actionable service improvements that reduce preventable maternal and perinatal mortality.

## Supplementary Information

Supplementary Material 1.

## Data Availability

All data generated have been included in the manuscript and supplementary files.

## Abbreviations

CRVS: Civil Registration and Vital Statistics
FCT: Federal Capital Territory
LMICs: Low- and Middle-Income Countries
LSPHCB: Lagos State Primary Health Care Board
MDR: Maternal Death Reviews
MDSR: Maternal Death Surveillance and Response
MPD-4-QED: Maternal and Perinatal Database for Quality, Equity and Dignity
MPCDSR: Maternal, Perinatal, and Child Death Surveillance and Response
MPDSR: Maternal and Perinatal Death Surveillance and Response
MSS: Midwives Service Scheme
NOQA: National Obstetric Quality Assurance
NPHCDA: National Primary Health Care Development Agency
OQA: Obstetrics Quality Assurance
SOGON: Society of Gynaecology and Obstetrics of Nigeria
UNFPA: United Nations Population Fund
WHO: World Health Organization
