# Supplementary material for: Tracking and appraising maternal and perinatal death surveillance and response implementation in Nigeria: a historical timeline and policy analysis
Source: Reprod Health. 2025 Nov 26;22:269. doi: 10.1186/s12978-025-02196-7 (PMC12751755; doi:10.1186/s12978-025-02196-7)
Supplement: Supplementary file 1 — Supplementary Material 1. [file 12978_2025_2196_MOESM1_ESM.pdf]

Supplementary file 1. MPDSR evolution in Nigeria search strategy

**Database / Source**	**Search terms / Strategy used**	**Filters applied**	**Hits retrieved**
PubMed	("maternal death review" OR "maternal death surveillance" OR "MPDSR" OR "MDSR") AND (Nigeria)	No date/language filters	14
Scopus	TITLE-ABS-KEY(("maternal death surveillance" OR "maternal death review" OR "perinatal death audit" OR "MPDSR") AND Nigeria)	No filters applied	14
Directory of Open Access Journals (DOAJ)	"maternal death" AND "review" AND Nigeria	No filters applied	19
Google Scholar	All in title: ("maternal death review" OR "MPDSR") Nigeria -- First 200 results screened	First 200 results screened	200
Grey Literature (WHO, UNFPA websites, NGO websites, Nigeria MoH, Google)	Manual search using terms: “MPDSR Nigeria”, “maternal death review Nigeria”, “perinatal death audit”	No filters applied	64
